# The Impact of the Gut Microbiome, Environment, and Diet in Early-Onset Colorectal Cancer Development

**DOI:** 10.3390/cancers16030676

**Published:** 2024-02-05

**Authors:** Rui Dai, Bridget N. Kelly, Amarachi Ike, David Berger, Andrew Chan, David A. Drew, David Ljungman, David Mutiibwa, Rocco Ricciardi, Gerald Tumusiime, James C. Cusack

**Affiliations:** 1 Department of Radiology, Massachusetts General Hospital, Boston, MA 02114, USA; 2 Harvard Medical School, Harvard University, Boston, MA 02115, USA; dberger@mgb.org (D.B.); achan@mgh.harvard.edu (A.C.); dadrew@mgh.harvard.edu (D.A.D.); rricciardi1@mgh.harvard.edu (R.R.); 3 Department of Surgery, Massachusetts General Hospital, Boston, MA 02114, USAaike@mgh.harvard.edu (A.I.); 4 Department of Gastroenterology, Massachusetts General Hospital, Boston, MA 02114, USA; 5 Sahlgrenska University Hospital, University of Gothenburg, 413 45 Gothenburg, Sweden; david.ljungman@surgery.gu.se; 6 Department of Surgery, Mbarara University of Science and Technology, Mbarara P.O. Box 1410, Uganda; dmutiibwa@must.ac.ug; 7 School of Medicine, Uganda Christian University, Mukono P.O. Box 4, Uganda; tumurald@gmail.com

**Keywords:** early-onset colorectal cancer, EOCRC, young onset, adenocarcinoma, gut microbiome, inflammation, diet, environmental exposures, birth-cohort effect, risk factors, clinical translation

## Abstract

**Simple Summary:**

Population statistics from recent years have demonstrated that rates of colorectal cancer among younger individuals have been increasing with alarming rates of mortality. This is contradictory to expectations given preventative clinical measures and suggests that there are factors that may cause greater rates of cancer in younger generations that are different than in older populations. Bacteria and other microbial organisms in the gut microbiome are crucial to human health, and research studies are beginning to demonstrate that disruptions to the gut microbiome are tied to recent increasing trends of colorectal cancer in younger populations.

**Abstract:**

Traditionally considered a disease common in the older population, colorectal cancer is increasing in incidence among younger demographics. Evidence suggests that populational- and generational-level shifts in the composition of the human gut microbiome may be tied to the recent trends in gastrointestinal carcinogenesis. This review provides an overview of current research and putative mechanisms behind the rising incidence of colorectal cancer in the younger population, with insight into future interventions that may prevent or reverse the rate of early-onset colorectal carcinoma.

## 1. Introduction

**Figure 1:**
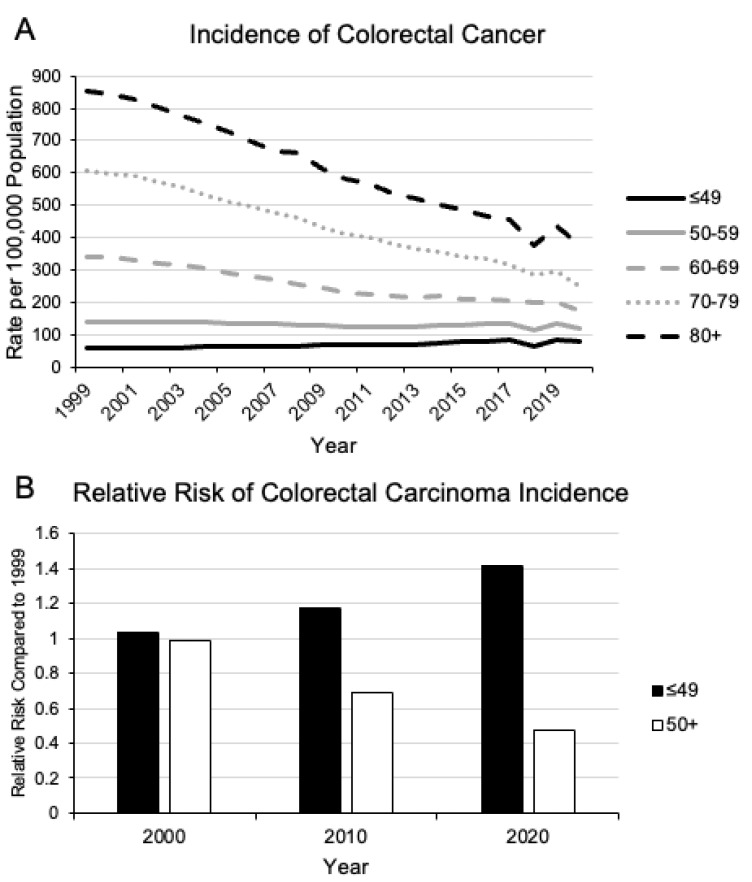
Change in incidence of colorectal carcinoma since 1999. Since 1999, there has been a steady decline in the incidence of colorectal carcinoma on a population basis (**A**). However, in patients younger than 49 years old, there has been a disproportionate increase in relative risk compared to the rest of the population (**B**). Data Source: https://www.cdc.gov/cancer/uscs/dataviz/download_data.htm (accessed on 6 December 2023) [10].

Colorectal cancer (CRC) is currently the third most prevalent cancer in the United States and the second most common cause of cancer-related deaths [1]. Traditionally considered a disease of populations over the age of 50, there has been a steady rise in CRC diagnoses in younger patients with greater rates of mortality in stark contrast to other age categories [2,3] (Figure 1). Population-based screening has decreased the incidence and mortality rates of traditional average-onset CRC (AOCRC), but that alone cannot explain the discrepancy in the population data. In individuals younger than age 50, the incidence of CRC has increased annually by 1–2% since the 1990s [4,5]. It is estimated that by 2030, the incidence of early-onset CRC (EOCRC) will increase by 90–124%, compared to the rate in 2010 [6,7].

Approximately 85–90% of CRC tumors are sporadic without specific heritable genetic factors [8,9]. Studies on germline genetics from CRC tumors, without known predisposition syndrome to CRC or inflammatory bowel disease, found that tumors from young patients have greater rates of pathogenic germline variants than that of AOCRC, which suggests that tumors from younger patients have greater somatic genetic mutations or epigenetic alterations induced by risk factors that are increased in the younger population [3]. In this review, we examine literature relevant to how intrinsic and extrinsic factors may impact the microbiome and their potential effect on the increasing rate of early-onset colorectal carcinoma.

## 2. Microbiome and Colorectal Carcinoma

From birth, different population cohorts are exposed to a wide range of different factors that impact their health and risk factors for disease. Individuals born at different time periods and locations have different exposures. Research examining the totality of these exposures for different populations have proposed a framework concept known as exposomes, whereby life-time exposures to different external and internal factors contribute to different risk factors of different birth cohorts [11,12]. This implies that because younger populations have been exposed to a different set of factors by a given age compared to the generation before them at the same age, these factors contribute to a different risk factor profile for different diseases. This may explain why the risk for CRC in the current young population is higher than in generations before. This is inherently different from what has conventionally been considered as a period effect, whereby an event at a particular time and location affects everyone equally [13,14]. The birth-cohort effect suggests that due to the cumulation of different life-time exposures, younger populations have different relative risks for various diseases compared to older generations. When examining the population statistics for different cancers, we find that this is true for CRC (Figure 1B). Younger birth-cohorts are at higher risk of CRC compared to older generations at the same age, and these risks theoretically will only accumulate and increase with age.

Previous studies have already found numerous birth-cohort associated life-style factors that are linked with increased rates of CRC, such as diet, sedentary lifestyle, smoking, and alcohol use [2,15,16]. Furthermore, these factors also all appear to directly impact the gut microbiome [17], which is composed of different populations of bacterial, viral, fungal, and protozoan species. The organisms that compose the gut microbiome are cumulatively greater in number than the number of cells in the human body, and could be considered as an “internalized external organ” with an ever more expanding role in the immunity and autoinflammation of the gastrointestinal system [18]. There is an irreplaceable symbiotic relationship between the microbiome and its host. It is required for homeostatic human health, but also contributes to human disease. For example, the gut microbiome is required for the production of vitamins and essential fatty acids not found elsewhere in the body [19]; conversely, pathologic changes in the microbiome are associated with increased inflammation and carcinogenesis [9].

An emerging body of research studies suggests that changes to the host microbiome are intimately tied to early-onset CRC carcinogenesis [20,21]. However, questions of what defines a pro-versus anti-carcinogenic microbiome, and of how a healthy microbiome morphs into a dysbiotic pro-tumorigenic one, remain largely unanswered. More research is needed to understand the mechanisms behind these processes.

**Figure 2:**
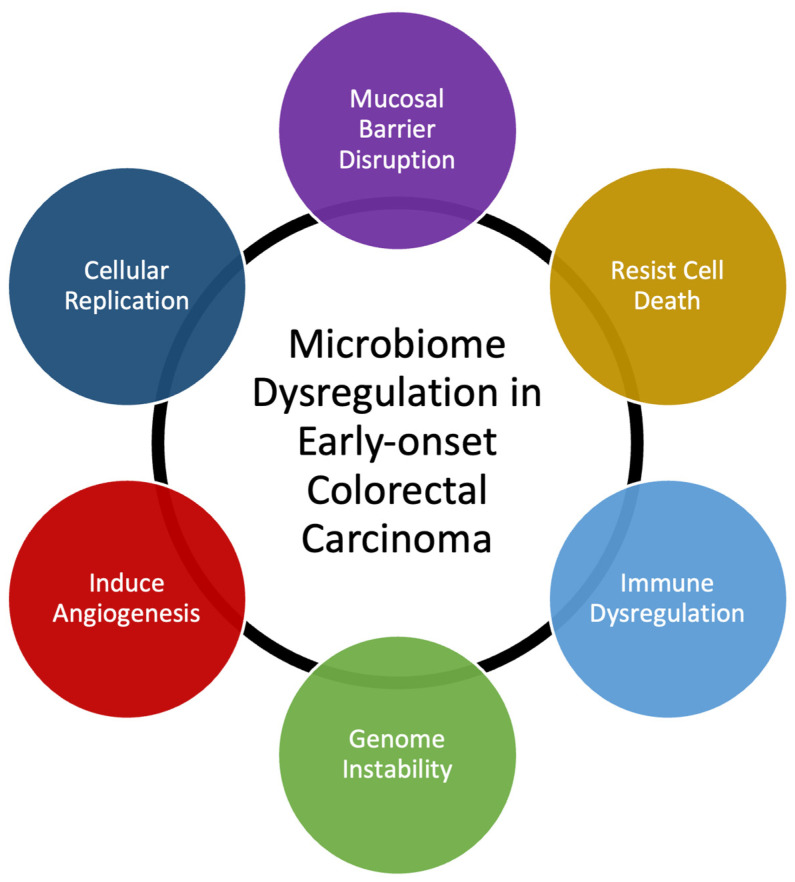
Summary of mechanisms that microbiome dysregulation may contribute to colorectal carcinogenesis and the increase in early-onset colorectal carcinoma.

Many bacterial species have been identified as directly responsible for autoinflammation and carcinogenesis [20,22]. Our own data demonstrate that T-cell infiltration and the expression of inflammatory mediators are similar in EOCRC and AOCRC, suggesting that an innate immune response may be requisite to colorectal carcinogenesis in general [21,23]. However, though the presence or absence of individual species may be essential to disease, changes to the composition of the rest of the gut microbial populations also appear to be necessary for its pathogenesis. Here, we provide a generalized overview of possible mechanisms behind how dysbiosis may induce early-onset colorectal carcinoma (Figure 2). Gut microbiome dysbiosis is broadly defined as an imbalance of the microbial ecosystem. At baseline, there are different microbial populations that inhabit different locations of the gastrointestinal tract. These microbial populations interact with each other and the human host. Dysbiosis occurs when the normal homeostatic balance is disrupted. The balance between protective versus harmful microbial communities and metabolism is likely central to understanding where imbalances result in tumorigenesis [24,25].

Microbial dysbiosis has already been proven to be crucial for many diseases and conditions of the gastrointestinal tract. For example, the sole presence of toxigenic *Clostridioides* (formerly *Clostridium*) *difficile* is not enough for the development of pseudomembranous colitis. In addition to the presence of toxigenic strains, microbial dysbiosis with overpopulation of *C. difficile* and disruption to other microbial species is required prior to the development of diarrheal symptoms and clinical disease [26,27].

More studies are needed to examine microbiome composition and shifts in microbiome components in response to different external factors: how the microbiome changes to external factors and how these changes drive tumor initiation, progression, and otherwise amplify other cancer risk factors. The connection between dysbiosis of the gut microbiome and the increasing incidence of CRC in young patients appears to have been hypothesized, but concrete evidence of the mechanistic interplay between gut dysbiosis and CRC in younger populations has yet to be published.

The human gut microbiome is composed of a wide range of bacteria, viruses, fungi, and protozoa, with up to 10^13^ to 10^14^ microorganisms and over 3 million genes, more than the entire human genome. Since the 1960s, it has been known that the carcinogen induction of CRC is intertwined with the microbiome [25]. Experiments with germ-free and conventional rats found that a known CRC carcinogen, cycasin, failed to induce cancer in germ-free rats opposed to conventional strains [28]. Subsequent experiments with other carcinogens found that *Escherichia*, *Enterococcus*, *Bacteroides*, and *Clostridium* bacteria were responsible for carcinogenesis by increasing the number of aberrant foci caused by the carcinogen 1,2-dimethylhydrazine [29]. The fecal transplant of patients with CRC to mice increased intestinal cell proliferation and tumorigenesis under the influence of carcinogen azoxymethane [30], which suggests that there is a causal relationship between the composition of the gut microbiota and development of CRC under different external stressors [31].

Studies on the gut microbiome in patients with CRC versus healthy individuals without CRC have identified different bacterial compositions between the two populations. When patients are analyzed along the adenoma–carcinoma sequence with metagenome-wide analysis, patients with adenoma demonstrated similar relative deprivations of microbial diversity as healthy individuals. However, patients with advanced CRC demonstrated higher microbiota genes in both absolute number and diversity than the healthy controls and patients with adenoma only [32]. This appears counter to studies analyzing fecal 16S rRNA genes that found patients with CRC had decreased overall microbial diversity via 16S rRNA sequencing, with specific lower relative abundances of specific bacterial species that may be protective against carcinogenesis [33]. This result could be due to technical differences between the studies, as one analyzed fecal 16S rRNA while the other used genome-wide association; however, it could also suggest that the gut microbiome is constantly in flux throughout the CRC developmental process, implying different microbiome compositions at dissimilar stages of disease progression.

Several shotgun metagenomic sequencing analyses have found a core set of colonic bacteria prevalent in patients with CRC and another set of anti-tumorigenic bacteria that are depleted in patients with CRC [34,35,36,37]. Although some common bacterial species have been found to promote CRC, including *Bacteroides fragilis* [38], *Escherichia coli* [39], *Enterococcus faecalis* [40], *Streptococcus gallolyticus* [41], and *Morganella morganii* [42], not every single clade in the specified species is carcinogenic. In fact, several of these bacteria are commonly found in the gastrointestinal systems of healthy individuals. In metagenomic analyses, the specific subspecies of the bacteria that cause inflammation and the associated toxigenic genes are not always specified. The mechanisms behind the role of each bacterial clade in carcinogenesis appear to be varied and distinct, but almost all seem to cause an inflammatory response in the enteric mucosal lining. Enterotoxigenic *Bacteroides fragilis* induces inflammation by producing IL-17 via T_H_-17 T-cells and γδT-cells [38]. In colitis-susceptible IL-10-deficient mice, the mono-colonization of polyketide synthase-expressing *E. coli*, which specifically produce colibactin, a polyketide-peptide genotoxin, had increased rates of colorectal malignancy [39]. The depletion of putatively beneficial probiotic bacteria in patients with CRC is less well studied compared to carcinogenic phyla, and the data are more conflicting, but notable species include *Streptococcus thermophilus* [43,44], several Lactobacillus strains [45,46], *Clostridium butyricum* [47], and *Carnobacterium maltaromaticum* [48].

In addition to bacteria, the gut microbiome is also composed of other microorganisms, including viruses [49] and fungi [50], that are altered in patients with CRC, though the data are relatively sparse and occasionally conflicting [31]. Excessive cytomegalovirus, John Cunningham (JC) virus, Epstein–Barr, and human papillomavirus have been identified in human CRC fecal samples [51,52,53]. Increased abundances of Malassezia and other fungi also appear to be associated with CRC [54]. Many of the different microorganism communities crosstalk and influence each other to create dynamic and mutable microbiome, which can contribute to the propagation of CRC [55].

## 3. Effect of Diet and Environmental Factors on the Microbiome

The influence of environmental extrinsic factors on the gut microbiome and subsequent effects on carcinogenesis remains a field of active investigation (Table 1). Since the early 1900s, it has been established that diet is one of the main contributors to changes in the gut microbiome [56]. The gut microbiome is generally stable over time under conventional circumstances, but significant dietary interventions have also been demonstrated to cause rapid changes over short amounts of time. A metagenomic analysis of fecal samples from 308 male participants without targeted interventions found that between-participant variation was consistently higher than longitudinal changes in the microbiome over 6 months [57]. With dietary intervention, however, targeted qPCR and 16S rRNA sequence analyses found that different bacterial blooms changed within 24–48 h of each intervention, particularly in response to indigestible carbohydrate fibers [58,59]. Longer-term chronic dietary changes in the gut microbiome can even impact the hosts’ offspring with clear generational effects over time. Bacterial populations that decrease after prolonged periods of low carbohydrate diets are not recoverable in several subsequent murine generations even after the reintroduction of the missing carbohydrates, requiring the reintroduction of the lost taxa in addition to replacing the lost dietary carbohydrates [60].

One of the most prominent representative analyses of differing diets and their impact on disease is the comparison between modern Western diets and rural agrarian diets. Compared to a rural agrarian diet, the microbiome under a Western diet has significantly lower microbial diversity [61]. The modern Western diet appears to increase specific populations of bacteria, which can produce metabolites that form gut microbial exposomes in the host body [62]. The production of *N*-nitroso compounds and hydrogen sulfide by specific toxigenic bacterial species in nondiverse gut microbiomes exert carcinogenic effects with DNA alkylation and genetic mutations of the gastrointestinal cells [63,64]. Increased rates of CRC development in mice that were fed a Western diet could be attributed to microbial dysbiosis. CRC progression was accelerated after a transplantation of feces from obese to nonobese mice, which could be blocked by continuous treatment with a mix of antibiotics, including ampicillin, vancomycin, neomycin, and metronidazole [65].

In addition to dietary fats and microbial-accessible carbohydrate fibers, the well-demonstrated impact of Western diet on the gut microbiome appears to be also due to a combination of red meat and processed pre-packaged foods. Red meat appears to promote the selective growth of certain bacterial populations through an excessive production of *N*-nitroso compounds and lipid peroxidation [66]. Ubiquitous in processed foods are emulsifiers that can act like detergents and increase the permeability of the mucosa, increasing bacterial movement across the epithelium and promoting inflammatory bowel disease even at relatively low concentrations [67]. This translocation is counteracted by some soluble plant carbohydrate fibers that inhibit bacterial adhesion and invasion in a dose-dependent manner [68]. The subsequent fermentation of the microbial accessible fiber also produces short-chain fatty acids, which have been demonstrated to regulate intestinal immunity and enhance the CRC treatment response [69]. Although the mechanism causing this phenomenon is largely unclear, it appears to be independent of bacterial growth since the most effective plant fibers seem to selectively enhance the growth of specific bacteria species.

Normally, gut bacteria metabolize dietary indigestible carbohydrates into short-chain fatty acids, such as butyrate, that can be absorbed into the systemic circulation to regulate immune cells [70], epigenetically decrease the rate of proinflammatory cytokine production [71], downregulate integrin to induce the apoptosis of some CRC cancer lines in vitro [72], and suppress carcinogenesis [73]. This process can be disrupted in the presence of many extrinsic factors, such as microplastics, nitrates, pesticides, and other chemicals, which result in disease. At the population level, countries with looser environmental regulations have seen a disproportionate rise in CRC over the last few decades, particularly in local regions with higher rates of pesticide use and/or air pollution [74,75,76]. Other studies have demonstrated increased serum-level pesticide levels in patients with CRC [77] and increased risk of CRC in populations with high exposure to pesticides [78,79]. Even at maximum residue levels tolerated by the European Commission and United States Department of Agriculture, pesticides have been demonstrated to alter the composition of the gut microbiome in both humans and animals [80]. Similar to dietary impacts on the gut microbiome, chronic exposure to pesticides, such as those containing arsenic, appears to drastically change the composition and metabolism of the gut microbial populations [81].

For microplastics, a model of polyethylene terephthalate (PET) digestion in a simulated gastrointestinal system demonstrated that in different parts of the colonic environment, microplastics are modified by indigenous bacterial species [82,83]. In turn, the addition of microplastics into the gut microbiome significantly changed the bacterial composition over 24–72 h at different points in the ascending, transverse, and descending colon of the simulated gut. In vivo studies have been difficult to perform due to inherent experimental limitations; however, microplastics have been linked to bacterial dysbiosis and intestinal irritation [84]. In female mice exposed to bisphenol A (BPA), there were significantly increased abundances of *Mogibacteriacae*, *Sutterella* spp., and *Clostridiales* bacteria [85,86].

The link between microplastics and CRC is less well established. An analysis of tumor biopsy samples from patients with CRC versus non-tumoral colon tissue from both patients with CRC and healthy controls found that the tumor tissue had a greater number of microplastic particles than the non-tumoral colon tissue from both CRC patients and healthy controls [87]. Microplastic particles in samples from the CRC patients (both tumor and non-tumoral colon tissue) were generally smaller than those in the healthy controls, and smaller particles tended to aggregate more than larger particles, though the difference was not statistically significant. However, aside from specimen analyses, a more direct link between microplastics and CRC has yet to be established.

## 4. Effect of Host Factors on the Microbiome

Outside of extrinsic factors that influence the microbiome, generational shifts in intrinsic factors, such as disease burden and general populational health, may also contribute to the rise in EOCRC. Diabetes, obesity, and smoking are well-established modifiable risk factors of CRC [88]. As the rate of younger patients with diabetes and obesity rises, the associated risks of these conditions on CRC have also been suspected to contribute to the rise in EOCRC [89,90].

Several recent studies have prospectively found increased risk of EOCRC in individuals with diabetes and obesity at the population level [90,91,92,93,94]. For every 10 kg increase in body weight above the self-reported baseline or ideal weight, there is an associated 8% increase in the risk of CRC [95]. The mechanism underlying the weight–CRC association is still unclear. Obesity has been linked to the dysregulation of the endocrine, immune, and metabolic systems, including excessive adipocyte deposition resulting in the preferential promotion of the proliferation and migration of colon cancer cells in tumor microenvironments [96,97]. Excess adipose tissue can also remodel the gut microbiome, such as by increasing the abundance of lipopolysaccharide endotoxin-producing gram-negative bacteria, resulting in the disruption of the mucosal barrier and low-grade chronic inflammation [98]. Subsequent experiments found that several bacterial populations appeared to contribute to obesity’s link with CRC, yet no concrete list has been determined due to the large variance in population characteristics and experimental models [99]. However, what is clear is that obesity promotes the development of bacterial and viral microbiomes associated with increased rates of CRC [100,101].

Host immune systems also appear intricately linked to CRC and microbial dysbiosis. The gut microbiome is closely tied to systemic inflammation, modulating numerous immune cellular populations throughout the body. One of the first lines of defense is the innate immune system that is governed by receptors, such as the Toll-like receptor (TLR), which recognizes pathogen-associated molecular patterns (PAMPs). Mice with an innate immune system deficient in TLR signaling are unable to recognize some PAMPs and have decreased rates of insulin resistance and adipose inflammation [102,103]. This appears to be due to macrophage infiltration into adipose tissue in response to the bacterial endotoxin activation of TLR.

The microbiome is crucial in training the human immune system, and the immune system, in turn, is responsible for maintaining the homeostatic balance or disruption of the microbiome into dysbiosis [104]. Increased microbiome-mediated colonic inflammation is associated with increased rates of DNA mutation. Mice deficient in TLR MyD88, therefore having suppressed innate immune systems with a limited ability to recognize PAMPs, were found to be immune from microbial dysbiosis-associated tumor progression [65]. However, localized immunosuppression can also be associated with increased rates of metastases. *Fusobacterium nucleatum* colonization increases proliferation immunosuppressive cells at pre-metastatic niches, which appears to be linked to increased rates of CRC metastases to the liver [105,106]. Additionally, immunodeficient mice are associated with increased risks of cancer, regardless of microbiome composition [107]. A homeostatic balance in the immune system also appears to be necessary for tumor suppression.

## 5. Practical Implications for Microbiome-Associated Prevention or the Treatment of Colorectal Carcinoma

Given the emerging evidence connecting microbiome dysbiosis and colorectal carcinoma, it appears imperative to reverse current trends that might be contributing to EOCRC. However, given the lack of cohesive targets, strategies to decrease microbiome-associated risks are still in the early stages. Different dietary interventions for individuals with varied bacterial populations resulted in various outcomes. Increasing dietary fiber can increase the microbial diversity, but only in susceptible populations [58,108].

Preliminary evidence using a probiotic mixture composed of *B. longum*, *B. bifidum*, *L. acidophilus*, *L. plantarum*, and various microbial-accessible starches was found to decrease the growth of transplanted murine colorectal cancer cells in vivo [109]. In mice with CRC induced by 1,2-dimethylhydrazine, probiotics composed of *L. acidophilus*, *L. paracasei*, *B. lactis*, and *B. bifidum* decreased the rate of adenoma-to-carcinoma progression when co-treated with 5-fluorouracil [110]. The mechanism behind this effect appeared to be the up-regulation of IFN-gamma and Granzyme B production, increased tumoral infiltration of NK and CD8+ T-cells, and promotion of Th1 CD4+ T-cells, which upregulate the body’s own immune system to attack the tumor cells [45,111].

However, these findings should be considered with caution as the perturbation of the microbiome for other cancer treatments has been less optimistic. Studies examining the effect of microbiome modulation on cancer treatment regimen have found that increased dietary fiber is associated with improved disease-free survival in patients receiving checkpoint inhibitor for melanoma, but this benefit is disrupted after the use of commercially-available *B. longum* or *L. rhamnosus* GG-based probiotic supplements [112]. Mechanistically, probiotics do not appear to repopulate the intestinal microbiome. Instead, they appear to interfere with the quorum sensing of existing bacteria populations, thereby competitively inhibiting their growth [113]. This may explain why probiotics interfered with the beneficial effects of high-fiber diet on microbiome changes in patients under checpoint inhibitor treatments.

Previous studies have found that the fecal transplant of CRC patients to mice resulted in increased carcinogenesis and tumor progression [30,114]. However, experiments examining whether fecal transplants can prevent or aid in the current treatment of colorectal carcinoma are relatively sparse. Studies on fecal transplants in combination with check point inhibitors for multiple solid malignancies and metastases have been promising [115].

Whether these interventions could impact the rising trend of EOCRC remains open to debate. Ultimately, current screening criteria for CRC may need to be further updated to reflect the uptrends in EOCRC. The 2021 US Preventative Services Task Force recommendations for adults with average risk of CRC was adjusted to begin screening at 45 years of age [116,117]. Similarly, the 2018 American Cancer Society (ACS) guidelines for screening adults with average risk of CRC were revised to begin at age 45, rather than age 50, using a high-sensitivity fecal occult blood test, a fecal immunochemical test, multitarget stool DNA assay, computed tomography, flexible sigmoidoscopy, or colonoscopy. However, given the increasing incidence of late stage EOCRC at time of diagnosis, the guidelines may warrant further modification [118].

## 6. Discussion and Future Directions

As the incidence of colorectal carcinoma continues to rise in a young population that is not routinely screened, it is important to uncover the mechanisms underlying the demographic shift. Given the low number of generations accounting for the populational shift, current available data suggest that the increasing EOCRC incidence is most likely due to external factors on the human body, rather than shifts in the human genome. In addition to the structural components of the human body, microorganisms populate the surfaces of external skin and intestinal lumen. Microbiomes of bacteria, fungi, viruses, and other protozoa are essential for human survival and thrive in a symbiotic relationship with their hosts. Disruptions to these relationships and shifts in microbiome compositions have clear implications on human health. The links between dysbiosis and carcinogenesis shown in cross-sectional studies appear irrefutable. However, definitions of what composes a “healthy” human microbiome are still not fully understood.

Several bacterial species have been linked with CRC, and the list continues to grow. However, external stressors may change not only the composition of the microbial populations, but also the behavior. In addition to analyzing the fecal contents of patients with and without EOCRC, we also need to understand how the microbiome functions in different stages of the intestinal tract, and how the microbiome changes in response to different stressors. In this review, we examined intrinsic and extrinsic factors that have been implicated in CRC through the gut microbiome. The next step is to investigate how these stressors change the behavior of the microbial populations.

Though the manipulation of the gut microbiome is compelling to prevent rising EOCRC trends, interventions should be implemented with caution and evaluated within the context of clinical trials. Given that we do not yet fully understand the cause of the disease or possible treatment-related changes, without strict control of experimental variables, the data may only challenge and delay knowledge of the disease and its mechanisms.

## 7. Conclusions

Early-onset colorectal carcinoma has been increasing at an alarming rate among individuals under 50 years old. The causes of this trend are not entirely clear, but evidence has emerged to suggest that environmental and populational changes in the microbiome play a vital role in colorectal carcinogenesis among young cohorts. In addition to elucidating the possible mechanisms behind this phenomenon, it is imperative that we further investigate possible ways to prevent and reverse the potential causes and implement clinical guidelines to screen younger patients to begin treatment at earlier stages of the cancer.

## Figures and Tables

**Table 1:** Summary of some factors that have been found to influence the composition of the microbiome.

Factors That Influence the Microbiome		
Host Factors	Environmental Factors	Dietary Factors
Diabetes	Alcohol	Fiber intake
Exercise	Microplastics	Indigestible carbohydrates
Genetics	Pesticides	Western diet and red meat
Immune health and immunosuppression	Other chemical exposures	Probiotics and fecal transplant
Obesity	Smoking	Processed foods

## Data Availability

The data presented in this study are available from the Centers for Disease Control and Prevention: United States Cancer Statistics repository at https://www.cdc.gov/cancer/uscs/dataviz/download_data.htm (accessed on 6 December 2023).
